# Staying with the trouble of networks

**DOI:** 10.3389/fdata.2022.510310

**Published:** 2023-01-26

**Authors:** Daniela van Geenen, Jonathan W. Y. Gray, Liliana Bounegru, Tommaso Venturini, Mathieu Jacomy, Axel Meunier

**Affiliations:** ^1^Collaborative Research Center “Media of Cooperation, ” University of Siegen, Siegen, Germany; ^2^Department of Digital Humanities, King's College London, London, United Kingdom; ^3^Center for Internet and Society, CNRS, Paris, France; ^4^Medialab, Université de Genève, Geneva, Switzerland; ^5^Aalborg University, Copenhagen, Denmark; ^6^Goldsmiths, University of London, London, United Kingdom

**Keywords:** critical data practice, feminist epistemologies, ethnomethodology, science and technology studies, data studies, algorithm studies

## Abstract

Networks have risen to prominence as intellectual technologies and graphical representations, not only in science, but also in journalism, activism, policy, and online visual cultures. Inspired by approaches taking trouble as occasion to (re)consider and reflect on otherwise implicit knowledge practices, in this article we explore how problems with network practices can be taken as invitations to attend to the diverse settings and situations in which network graphs and maps are created and used in society. In doing so, we draw on cases from our research, engagement and teaching activities involving making networks, making sense of networks, making networks public, and making network tools. As a contribution to “critical data practice,” we conclude with some approaches for slowing down and caring for network practices and their associated troubles to elicit a richer picture of what is involved in making networks work as well as reconsidering their role in collective forms of inquiry.

## 1. Introduction

Over the past few decades network analysis, visualization and other associated practices have become prominent in many areas of culture and society. This is commonly attributed to the growing availability of data with relational qualities (e.g., data generated by the web and social media) and research software to explore and analyze such data. Network analysis and graph visualization software tools have broad and diverse user bases spanning many different fields. At the time of writing, the paper that describes Gephi's implementation (Bastian et al., 2009), one of these software tools, has been cited in over 9,800 publications, most of which have used the tool as a research instrument for the visual exploration and analysis of relational data.^1^ Gephi is one of the tools that several of us have been involved in developing and using for teaching, research, and collaborative projects. These different projects serve as the basis for the following discussion of network practices and the troubles that may accompany them.

The field of social network analysis (cf. Marin and Wellman, 2014) constitutes only one of many areas in which networks are used as intellectual devices or graphical representations. Network practices are also prominent in the natural sciences to study, for example, biological and physical phenomena. Network analysis (i.e., the analysis of relational data) and network visualization (i.e., relational data spatially mapped as network graph) are also increasingly used beyond academia, in areas such as journalism, activism, policy, art, and marketing. Moreover, network maps as graphical representations are an important part of online visual cultures (e.g., Galloway, 2012; Munster, 2013). They circulate online not only as scientific diagrams, but also as illustrations, backgrounds, logos, dashboards, web interactives, storytelling devices and other visual materials.

As they are translated and used in different settings, methods of network analysis and visualization may also become detached from the circumstances, societal contexts and communities in which they were created (cf. Latour, 1986; Haraway, 1988; Ruppert et al., 2013). There are now many different groups of network practitioners,^2^ often with different histories, conventions, routines, and understandings of the methods they use and the outputs they produce (cf. Jacomy and Jokubauskaite, 2022). In order to attend to various ways of working with networks in different settings, we will examine three cases of trouble from our work, which foreground practices through and contexts in which tensions might arise. These cases draw upon our collaborations around the Public Data Lab, a cross-institutional, interdisciplinary network involving several research centers with which we are or were associated, including the médialab (Sciences Po, Paris), the Digital Methods Initiative (University of Amsterdam), the Department of Digital Humanities at King's College London, the Center for Internet and Society (CNRS, Paris), the TANT Lab (Aalborg University, Copenhagen), the Collaborative Research Center Media of Cooperation (University of Siegen), and the Datafied Society research platform (Utrecht University).^3^

In this article, we propose learning from and “staying with the trouble” (Haraway, 2016) of network practices as a contribution to the fields of critical data studies (e.g., Iliadis and Russo, 2016) and critical algorithm studies (e.g., Gillespie and Seaver, 2015; Kitchin, 2016), examining computational procedures and data as “sociotechnical assemblages.” That is, informed by perspectives in science and technology studies (STS), data and algorithms are seen as embedded in societal situations, shaping and being shaped by social and cultural practices, values, norms, and discursive-material modes of organization (Ibid.). Recent work in this area calls for more empirical examinations of how specific data and algorithmic practices are involved in the composition of knowledge, the production of economic assets, political decision-making, and everyday life (e.g., Seaver, 2017; Dencik, 2020; Rettberg, 2020). Following feminist STS scholar Donna Haraway, Jill Walker Rettberg (2020) proposes “situated data analysis” as a methodological stance that encourages researchers to explore and report on the circumstances that informed the generation and processing of data. Others have introduced “data journeys” (Bates et al., 2016) as a methodological approach for tracing “the life of data” and surfacing data frictions (Bates, 2018), as well as “data diaries” to reflect on how data “co-constitute a given spatial situation” (Tkacz et al., 2021, p. 2). This paper aims to contribute to such research approaches by suggesting ways of accounting for and reflecting on network practices.

In the following section, we introduce our understanding of trouble as an entry point to understanding and reflecting on how different communities of practice work with networks (cf. Garfinkel, 1967; Haraway, 2016). In doing so, we approach network practices as “matters of concern” (Latour, 2004) and “matters of care” (de la Bellacasa, 2017). Treating network practices as matters of concern and care calls for attentiveness not just to the theoretical and methodological frameworks that inform these practices, but to their overall sociomaterial organization, including the diversity of human and non-human actors involved in making them work. Situating network practices may open up space for doing things differently, as we return to at the end of this article with reference to “critical data practice” (Gray and Bounegru, 2021) and “critical technical practice” (Agre, 1997). Such approaches may encourage network practitioners to attend to how their practices are mediated through critical encounters with relational data and the digital technologies and infrastructures that facilitate and frame their creation, analysis, and visual representation (cf. Gray et al., 2016; van Geenen and Wieringa, 2020).

In the following sections, we discuss troubles related with networks “in the making” (Latour, 1987, p. 4) (in section 2.1), and with communicating networks to broader publics or “making networks public” (cf. Latour and Weibel, 2005) (in section 2.2). The examples we draw these kinds of trouble from are derived from our diverse engagements with the journalistic context: On the one hand, we studied evolving journalistic network practices through interviews and fieldwork exploring and mapping the field of digital journalism, investigative reporting, and data journalism. In this context we observed that networks can serve as discovery devices, accompanying journalists' investigations but are not necessarily shared as narrative devices to make findings public. This can be due to concerns about the representational fidelity of network maps vis a vis fields and actors under investigation, as well as interpretive issues associated with such network visuals. On the other hand, we describe troubles we encountered sharing networks created as part of our own research with journalist colleagues for publication as part of their investigations. This translation process was marked by tensions between aspirations for accessibility and newsworthiness and concerns about taking network maps out of context and as self-evident diagrams from which broader societal implications could be derived. We discuss issues that arouse in this course with collective sensemaking through networks, and especially, the need to find shared modes of accounting for the analytical work conducted and possible interpretative ambiguities encountered in this process. As a third area of trouble, we turn to how software tool development and design decisions (in section 2.3) play a role in shaping network practices, focusing on tensions between exploratory and explanatory engagements with network maps.

In section 3, we consider two complementary approaches for slowing down and attending closely to what is involved in the network practices that can be encountered in various field(s): a prototype “Fieldnotes Plugin” for Gephi and a series of protocols for observing and documenting network practices. We do not conceive of these approaches as general “best practices.” Rather, we understand and present them as encouragements to identify, acknowledge, and situate the diverse ways in which network analysis and visualization are practiced, and thus, networks are put to work.

As we discuss below, our purpose in surfacing troubles that arise in network practices, then, is not just to propose new solutions to these troubles, but to learn from them and what they may tell us about the changing roles of network practices in culture and society. While this article is grounded in social and cultural research, we hope that the invitation to dwell with, delve into and document trouble may be of relevance to researchers and practitioners in other fields in reconsidering how we know with networks and in contributing to potential alternative ways of working with them in collaborative settings.

## 2. Learning from trouble

In this article we consider “trouble” as a generative concept to reflect on the diversity of existing network practices, drawing inspiration from Haraway (2016) recent work in this area as well as Harold Garfinkel's practice theory (Garfinkel, 1967, p. 36–38). Garfinkel (1967) *Studies in Ethnomethodology* were concerned with the “ethnomethods” of practitioners: the everyday practices performed and articulated by people in professional and mundane settings. Garfinkel traced and “made trouble” to make explicit the tacit conventions that frame social interactions and order, particularly in workplace settings (Ibid., Chapter 6, in collaboration with Egon Bittner). Garfinkel's praxeological endeavor builds on the premise that “activities whereby members [of specific social groups] produce and manage settings of organized everyday affairs are identical with members' procedures for making those settings account-able.” That is to say, “observable-and reportable, i.e., available to members as situated practices of looking-and-telling” (Garfinkel, 1967, p. 1). In other words, in ethnomethodology, “account-ability” denotes and deals with practitioners' modes of accounting for and giving account of their professional practices, including those working with and on complex data-intensive approaches and computational or algorithmic techniques (Neyland, 2016, p. 55).

Inquiring into the situatedness of network practices as emerging from particular shared and cooperative ways of doing (i.e., “ethnomethods”) we follow the notion that the social is enacted in and needs to be studied through “practical accomplishments” (Garfinkel, 1967, p. 9). We take troubles encountered in network work, in academia and beyond, as moments to document and reflect on different social, cultural, and technical aspects of these practices building on our engagement with different communities of practice. “Trouble as a method” has also been taken up in science and technology studies (STS), where discursive-material frictions and controversies are used as a way of investigating and acknowledging different knowledge practices (e.g., Latour, 2004; Venturini, 2010). Trouble can serve as a prompt to situate knowledge practices (Haraway, 1988). Like Rettberg (2020), we build on Haraway's call to problematize disembodied knowledge claims in the generation, analysis and visualization of data. Following feminist STS approaches, we argue that data can be viewed not just as representations of the world, but also as infrastructures which play a role in shaping social organization (e.g., Gray et al., 2018; D'Ignazio and Klein, 2020; Rettberg, 2020).

Network analysis has a history which draws upon insights and approaches from various epistemic cultures (Knorr Cetina, 1999), from graph theory to social theory, which are inscribed into and mobilized by network tools (Rieder and Röhle, 2017). Addressing network troubles, then, is not just a matter of optimization or bug fixing [cf. introduction to *Staying with the Trouble* in Haraway (2016)], but an “empirical occasion” (Marres, 2013) to make visible the stakes, actors, and relations involved in network practices. This may contribute to the cultivation of what we call, after Haraway, network “response-ability” (2016, p. 105). We envisage “network response-ability” as an invitation to attend to different ways of working with networks and their associated contexts, cultures, genealogies and situations, and for making space for different ways of doing things in everyday exchanges and collaborations. Pursuing network response-ability implies reflecting on and pluralizing engagements with methods of network analysis and visualization in society.

### 2.1. Who and what are networks for? Troubles with making and using network graphs in journalism

Our first case study draws on research conducted between 2014 and 2019 on evolving network practices in journalistic knowledge cultures and communities. Those we talked to were aware of and involved in journalistic experimentation with networks going back to the 1990s, first with their own manually compiled databases, and later through access to information requests, data “dumps” and data “leaks.” Another growing source of data was “born digital” data (Rogers, 2013) from online platforms and the web. While the first decade of the 2000s saw concerns amongst journalists about data quality (as compared with manually compiled data or data sourced from public institutions), the rise of digital investigations around the 2016 US elections has been considered a turning point for re-using digital data (Gray and Bounegru, 2019). This period has also seen the rise of collaborations between researchers and journalists around the consequences of digital data, platforms, and algorithms in collective life (Bounegru and Gray, 2021).

**Figure 1:**
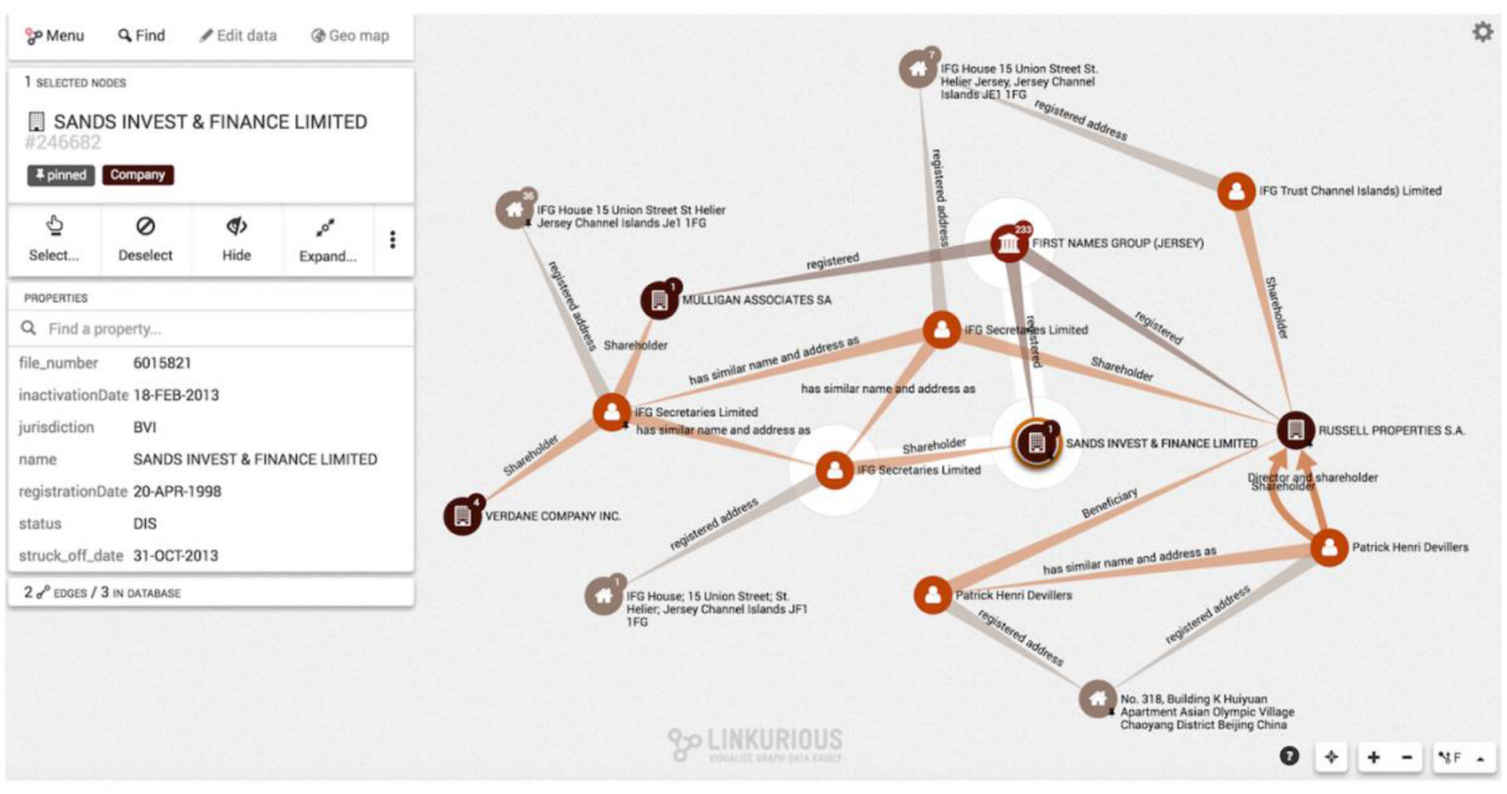
Network visualization with Linkurious tool from 2015 Panama Papers project showing different kinds of legal and financial relations between companies, addresses and people associated with them.

Several of us have conducted empirical research on the use of networks in journalism, including as *narrative devices* (Bounegru et al., 2017). This includes content analysis, field work and interviews with journalists on their network practices.^4^ While networks have become visual emblems of digitally mediated innovation in data and computational journalism, we found that they were also commonly used as *discovery devices* that would not be shared outside of investigative teams. For example, Paige St John's (The Pulitzer Prizes, 2011) winning investigations into the property insurance industry in Florida used networks to identify lines of investigation. Yet, these networks were not published and there was not often sign of them in the final stories. According to those we spoke to, networks were commonly used to explore “hidden” connections and “shortest paths” between entities (e.g., people or companies) in the context of investigative reporting and data journalism. While there were comments amongst practitioners in 2014 that networks had not yet had their “breakthrough moment,” they have since been used in prominent investigations such as the 2015 Panama Papers project (Figure 1).

In our interviews, journalists mentioned issues with relational data, including their non-availability and high costs.^5^ For example, investigators looking into company ownership often ran up against a lack of data on “beneficial ownership” - or who “really” owns and benefits from the control of legal entities aside from the “nominal” owners listed on official registers. There were questions around the “representativeness” of data. Some journalists took a piecemeal approach to assembling network databases, adding one element at a time (e.g., “went to school with,” “worked at the same place as”), which provided a rich set of perspectives, but left them wondering which connections were most relevant (“Is it that people went to kindergarten together or that they are Facebook friends?”) and which may have been missed. The time and costs of such manual “data work,” were also a source of concern, next to prohibitive costs and dubious ethics of tools like IBM i2 and Palantir's platform and the technical knowledge required (e.g., Cypher queries in Neo4J).

Some journalists were concerned about the “general uselessness of network diagrams,” including the danger of “proving what you already know,” showing “spurious” connections, or associations that are difficult to substantiate. One interviewee commented that while journalists had worked for decades to familiarize readers with formats such as interviews, polls, surveys, statistics, network conventions are still not common, and the time required to unpack these for readers is prohibitive.

As well as understanding journalistic network practices in relation to established conventions of social and network sciences (Anderson, 2018), one might take these concerns as an indication of other styles of data work and knowledge cultures which deserve recognition (Bounegru and Gray, 2021). Instead of focusing on quantifying relations or measuring the structure of networks, journalists were often more interested in tracing and investigating relations, in conjunction with other reporting methods. Their primary interest was to use networks as devices to combine heterogeneous, incomplete and partial data from a variety of sources (e.g., leaks, disclosures, web scrapes) in order to trace a link between different entities which would then provide starting points for investigations. Slowing down and spending time with these network troubles suggests how the context of journalists' work might differ from other network practitioners, and how they may use networks not predominantly for measurement and calculation, but rather as discovery devices or narrative devices incorporated into digital investigations and interactive stories (Bounegru et al., 2017; Venturini et al., 2018). Collaborations with network data and visuals may be enriched by not too quickly assuming the interests, assumptions, and practices of those involved.

### 2.2. What do networks mean? Trouble interpreting and reporting on the Dutch Twittersphere

The second case study concerns the translation of our own network practices into collaborations and outputs for broader publics. We examine challenges that can occur when network outputs are separated from their making, or the preceding exploratory and analytical work (cf. van Geenen and Wieringa, 2020). In 2016, researchers from the Datafied Society research platform embarked on an exploratory study of Twitter as a medium for everyday communication and information exchange and herein the role of users' geographical location and local engagement.^6^ The empirical starting point was a data sample of more than 7.6 million tweets containing a 2 week “snapshot” of the Dutch-speaking day-to-day communication, collected between 4th and 18th September 2016.^7^ Network analysis was used to identify different communities based on their political and professional interests and various ways of using the platform (van Geenen et al., 2016; Wieringa et al., 2018a).

**Figure 2:**
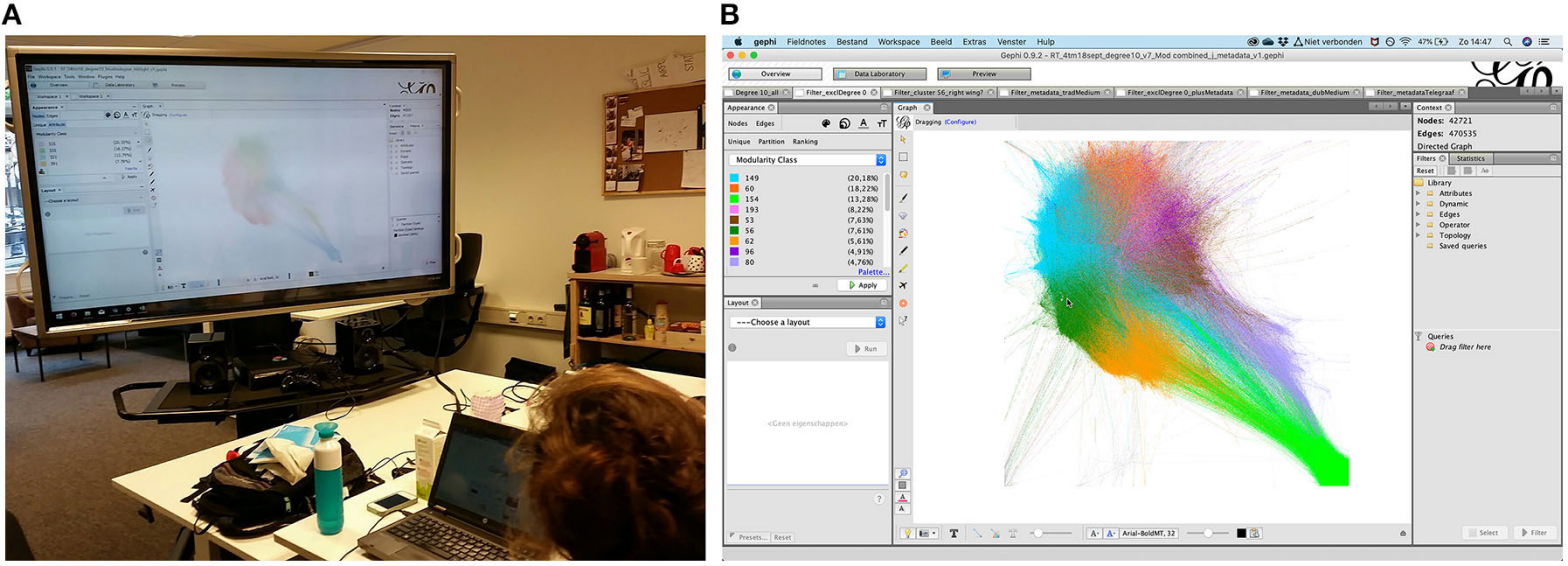
**(A, B)** Exploration and mapping of Dutch-speaking Twitter users as part of a collaboration between media researchers and journalists conducted by Daniela van Geenen and Maranke Wieringa at the office of the Datafied Society research platform. The photograph shows the construction of the retweet network in August 2017 on a large screen (left). The screenshot zooms in on the spatialized and filtered network graph of the same retweet network in Gephi's interface with the cursor pointing to the politically right-wing cluster (community 56) (right).

One part of the empirical investigation focused on users' “framing” practices on Twitter, the ways in which these users either approvingly or disapprovingly share others' content, as part of their political activities on the platform (Wieringa et al., 2018a). We used Gephi and its “ForceAtlas2” layout algorithm (Jacomy et al., 2014) for an initial exploration of the retweet practices of Dutch-speaking Twitter users, mapping these interactions as edges constituting a retweet network. The spatialization algorithm chosen for this mapping was specifically designed for visual analysis of relational data in Gephi allowing an on-screen spatialization and clustering of the network graph based on the simulation of simultaneous attraction forces between edges and repulsion forces between nodes (Ibid.). The clustering of the network graph was emphasized by its partitioning through color based on modularity community detection (Blondel et al., 2008). The graph was filtered in Gephi (i.e., nodes with ten or more connections) to focus only on the most retweeting and retweeted accounts (around 42,700 nodes and more than 1.3 million retweets) (cf. Wieringa et al., 2018b). Looking at this filtered graph and questioning the position of the nodes and the clusters that they formed (cf. Figure 2A), in combination with a preliminary qualitative encoding of the Twitter accounts that these clusters consisted of (e.g., based on these users' Twitter “bios”), we were able to start identifying several “political topic-communities” (Wieringa et al., 2018a).

In the subsequent analytical work conducted with an interdisciplinary team,^8^ we substantiated our exploratory efforts and developed the methodological approach to analyze the complete two-week “snapshot” of Dutch-speaking retweet behavior, which drew on a combination of quantitative and qualitative methods: Next to calculating modularity for and applying this “sociometric” principle to the sample of relational data and its mapping (cf. Newman, 2006 in Jacomy et al., 2014), we computed additional metrics for all clusters in the retweet network. These metrics included the clusters' density (of edges, compared to the number of possible edges between nodes in a cluster) and the PageRank (Page et al., 1999) of the nodes within these clusters, that is their centrality or “relevance.” We combined this analysis with more extensive close reading of accounts' profile information (i.e. “bios”) and the media references (hyperlinks) these users shared, focusing on accounts in two particular clusters of the retweet network: that is, accounts in (1) the with more than 40,000 accounts largest cluster featuring accounts that could be classified as representing center-left political positions, and (2) a cluster consisting of around 10,000 nodes representing accounts that, based on their profile information, adhere to (far-) right political ideas. We identified and compiled tweets with references to traditional and online-only media shared by the accounts in these two clusters,^9^ in order to identify the modes of framing these users applied. Part of the methodological setup and triangulation was the encoding of a representative sample of such tweets, distinguishing between affirmative, oppositional, and semi-endorsing stances toward the shared media references [see Wieringa et al. (2018a) for methodological elaborations].^10^

Our preliminary observations aroused the interest of Vrij Nederland, a Dutch weekly journalistic magazine. The ensuing collaboration, however, ended up being a kind of “tug-of-war” in making sense of our networks with others, between a demand for newsworthy and accessible findings and a need for interpretative nuance and methodological explanations. For instance, taking the clustering of accounts not as a definitive classification of communicative user behavior and political stances, but leaving space for interpretative ambiguities and doubt in this encoding and analytical process constituted an area of conflict. Frictions in the collaboration derived in part from the separation of our analytical work and the journalists' investigative process (data analysis and content production did not go hand in hand, as discussed by Smit et al., 2014). The fact that the project started off with certain reporting hypotheses—i.e., the idea that left and right groups should be distinguishable on Twitter—complicated the collaboration even more, as our findings showed no straightforwardly identifiable distinction between right-wing and left-wing communication patterns. Both the graphical representation of the network and the graph metrics indicated that, while a cluster of users was clearly identifiable for the right (cf. the dark green community 56 displayed in Figure 2B), no clear-cut cluster existed for the left.^11^,^11^ Instead, we found that the ways in which users shared and framed content were more relevant: for example, “cherry-picking” and commenting on certain parts of the content before spreading it (Wieringa et al., 2018a). These “curation” practices were common for both right-wing and, what we termed, “leftist” users (Ibid.). Qualifying these media practices of curation seemed more relevant to us than focusing on identifying opposing political communities.

Moreover, the publication of our network maps made us realize that the public may mistake them as a representation of the whole “Dutch Twittersphere,”^12^,^12^ rather than taking them as a snapshot of a sample of highly active Twitter users. Detached from its original methodological context, the network visualization might not just lose needed contextualization, but also take on new meanings and implications (cf. the discussion in van Geenen and Wieringa, 2020 and Jacomy, 2021, p. 27–50). Interpretability is not inherent to the network visualization; it depends on who is interpreting: their perspectives, expectations, knowledge about what is mapped, and familiarity with the used methods (cf. Ruppert and Scheel, 2019). Traditionally, sociological approaches to data draw on collective “data critique” sessions (Gießmann and Burkhardt, 2014), in which everyone involved in the investigation discusses and compares the analysis of the data against an examination of the circumstances through which the data were produced. Collective sensemaking of relational data with other practitioners could benefit from collaborative in-depth “readings” of the data and the methods used to approach them.

Eventually, information about the research process was dropped in the final article (cf. Broer and Ostendorf, 2018): the journalistic publication lacked space (especially in the paper version) to account for and report on our methodological considerations and the conducted interpretative work, which meant we had to publish our methodological account elsewhere (Wieringa et al., 2018b). This experience emphasized that bringing network visuals into new settings should not be taken for granted. On the contrary, “everyday engagements” (Kennedy and Hill, 2018) with networks require additional effort and care in sharing them, making sense with them and making them public.^13^,^13^

### 2.3. What should network software do? Trouble in the design of the MiniVAN tool

The question of how practitioners create and make sense of networks guided one lively workshop on the MiniVAN project (Public Data Lab, 2018)^14^,^14^ that took place in November 2018 in London. The MiniVAN tool originated from previous work on Gephi and other tools at the médialab, Sciences Po.^15^,^15^

**Figure 3:**
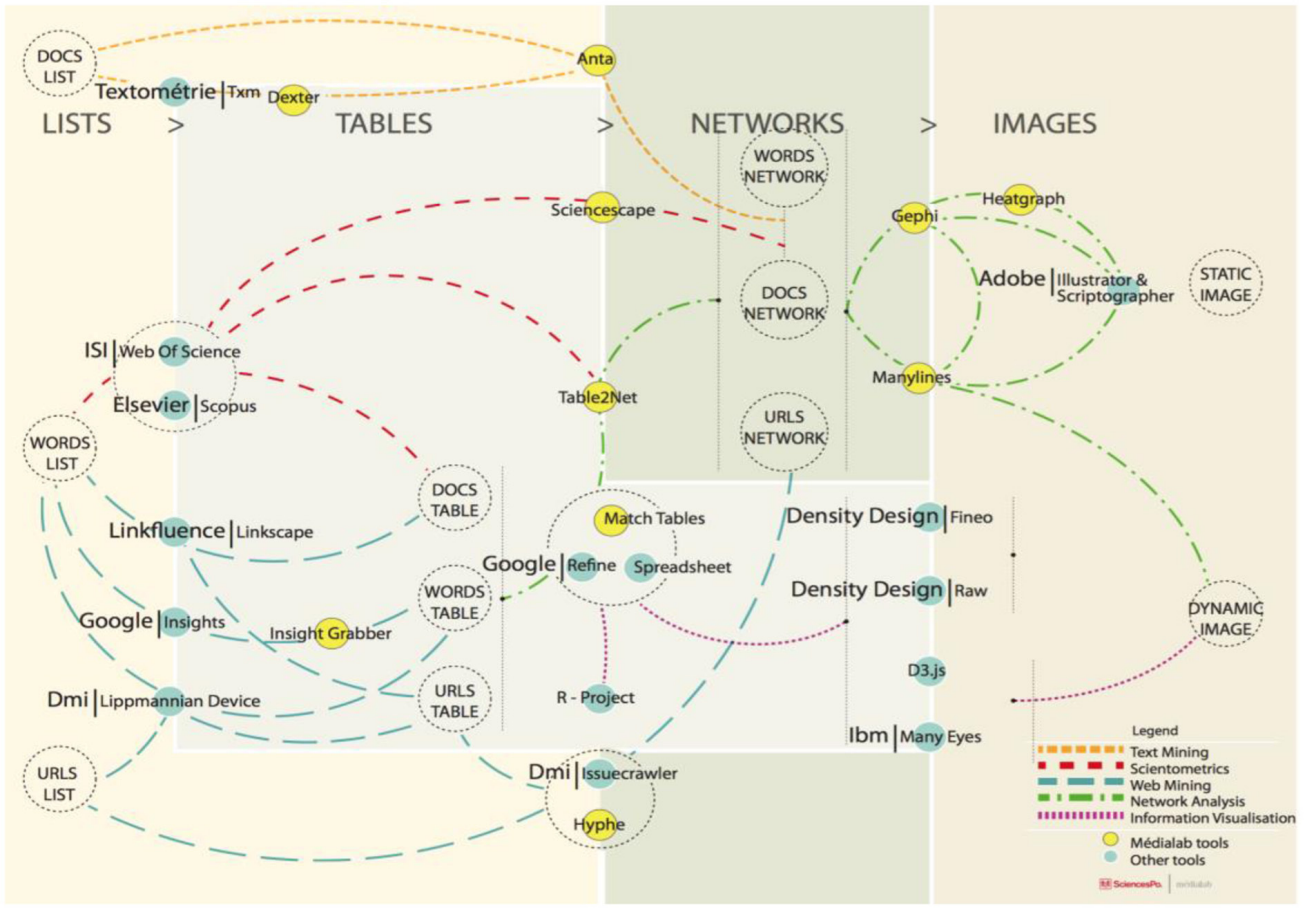
Diagram of network research processes using digital methods, designed at the médialab (Sciences Po), and presented at the MiniVAN workshop in London. The diagram shows different kinds of research tools, objects, and processes in research activities of the lab.

MiniVAN started out with the ambition to be an “easy-to-use tool that will support non-specialist social scientists in the visual analysis of their networks and in the online publication of their results” (Public Data Lab, 2018). As such it was designed to operate alongside other tools (see Figure 3): its original intended use scenario was to start with a network map prepared in Gephi and then use MiniVAN to publish the network online where it could be freely explored by anyone. A tension between publication and exploration, however, came into focus during the workshop, when the users asked about the possibility to add to the tool a function to publish network maps as widgets embeddable into external web pages.^16^,^16^ This demand sparked a heated discussion about MiniVAN's design premises.

The MiniVAN tool had initially been designed as a user-centric information system dedicated to a single network visualization that the reader could browse to access different, complementary views. The sensemaking process was envisaged in terms of navigational experiences within the MiniVAN tool. By contrast an embed function would be oriented toward publishing network maps alongside texts and other materials—such that the sensemaking process was envisaged to happen outside of MiniVAN, within a page explaining what to observe in each embedded visualization. An embed function could, thus, support an explanatory engagement instead of the initial exploratory intent. This demand for explanatory visualizations is perhaps not surprising, since Gephi is mainly a tool for exploration that externalizes the explanation of network visualizations to their publication, which is MiniVAN's main purpose. What is surprising is how entangled the exploratory paradigm turned out to be with technological constraints.

While everyone agreed that an embed feature could be valuable, the development team of MiniVAN had hitherto focused on navigation rather than on the publishing of curated views of a network. The possibilities of tool development were constrained by technological affordances, notably because embeds happen outside the boundaries of the tool. Creating embeddable widgets would complicate maintenance and further changes to the tool. Discussions highlighted how MiniVAN and associated projects were dependent on the evolution of programming languages (i.e., Java), web technologies (i.e., HTML5), graphics libraries, and other elements.^17^,^17^ If MiniVAN's initial design prioritized exploration, it was also because such a choice minimized technical maintenance problems.

**Figure 4:**
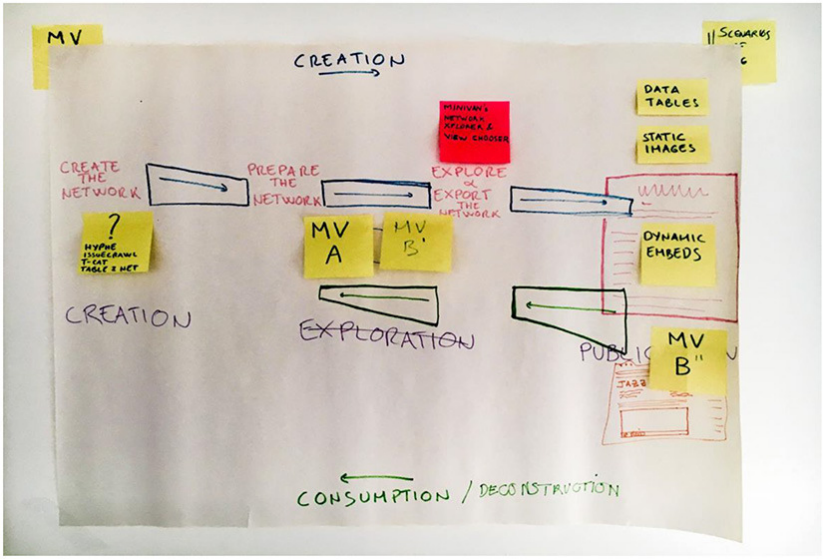
Diagram of network research process from MiniVAN workshop in London (November, 2018). The diagram shows different ways of creating and working with networks and associated interface features.

Was an embed function desirable enough to justify the effort—also promoting account(-)ability in network practices? In order to address this, the workshop sketched out various workflows and processes defining network practices, from “creation” to “exploration” to “publication” (Figure 4). We invited researchers, journalists, activists and others to talk about and bring visual or material artifacts exemplifying their network practices, and found that they were very different, including: working with graph databases, but not necessarily network graphs; exploring networks, but not necessarily publishing them; using coding notebooks to document network practices. While some were interested in the narrative capacities of data visualizations, many had yet to experiment with the capacities of networks for telling stories, including stories that provide information on how network maps were constructed. The workshop format was vital in making these practices visible to the developers, and in showing to the users the effort in design and coding necessary to answer their needs. The activities associated with the MiniVAN project thus surfaced friction, differences, and concerns regarding how the process of working with networks could be partitioned, modeled, and inscribed into tools. These troubles shed light upon a plurality of different ways of working with relational data, some of which were very different to our own conventions and expectations. Thus, the workshop surfaced a diversity of network practices involving particular technical infrastructures, interface features, knowledge cultures, and resources for development and maintenance, which may be taken into consideration in software design processes.

## 3. Discussion: How to “stay with the trouble” of network practices?

Prompted by the troubles discussed above, we turned toward a “situated” understanding of network practices.^18^,^18^ As we know from research in STS and associated fields, all knowledge practices are socially, culturally and historically situated (e.g., Latour and Woolgar, 1979; Latour, 1987; Haraway, 1988; Knorr Cetina, 1999). That network practices can be situated is therefore hardly surprising. What interests us is whether and to what extent accounting for this situatedness might modify the way we work with relational data in our research, teaching, and tool development.

While scientific knowledge is often presented as a matter of fact, following Latour (2004), we are interested in considering networks as “matters of concern.” Moreover, we advocate caring for (cf. de la Bellacasa, 2017) the multiplicity of social, cultural, epistemic, methodological, organizational, representational, discursive-material and technical aspects that influence the knowledge that can be obtained and conveyed with them. Such attention for practices of care and maintenance—as opposed to innovation and invention—increased only recently in STS and the philosophy of technology (e.g., Mattern, 2018; Russell and Vinsel, 2018; Vinsel and Russell, 2020). Following recent work in critical data and algorithm studies (e.g., Bates et al., 2016; Tkacz et al., 2021), we are interested in how specific situations could influence the inquiry into relational data. We addressed the need to pose these questions elsewhere, advocating “critical data practice” (Gray and Bounegru, 2021) and the “critical affordance analysis” of our research instruments (van Geenen, 2020). Our interest lies in how attending to and specifying the settings and situations of network practices may open up opportunities for interdisciplinary and trans-institutional exchanges, including for participatory research and “collective inquiry” (Gray et al., 2022). Particularly in light of recent work recognizing that expert institutions do not have a monopoly on how knowledge claims can be validated (Marres and Gerlitz, 2018), there may be other ways of responding to and staying with the multiplicity of network practices than through correction and conventionalization. In the following paragraph, we turn to some of the ways through which network practices can be accounted for that we have been developing in the context of our research, teaching, and collaborations.

### 3.1. Two sides of “account-ability” through a software plugin and an observation protocol

**Figure 5:**
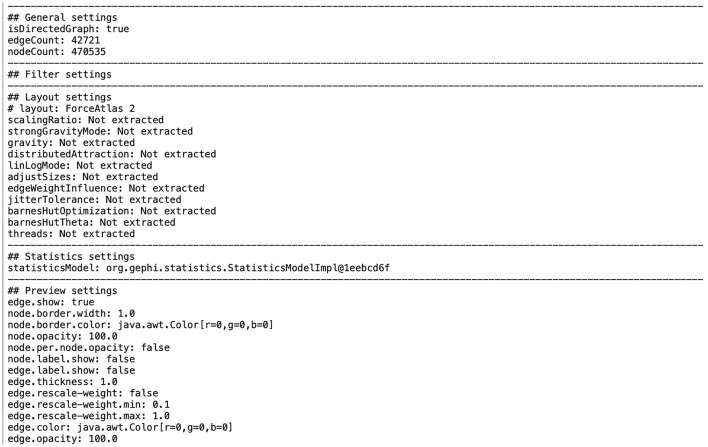
Screenshot of a settings text file created with the Gephi Fieldnotes Plugin summarizing basic details of the network graph and software settings and operations applied to it.

Spurred by working with Gephi in collaboration with various civil society actors and media organizations, researchers associated with the Datafied Society research platform created an operational prototype “Fieldnotes Plugin.” This plugin was developed in order to capture and document the various steps taken to generate a network visualization with Gephi (Wieringa et al., 2019). Running the plugin generates a snapshot of the network consisting of a graph file and a text file, which documents basic information on the network graph and on some of the applied settings (see Figure 5). This mode of accounting has affinities with approaches to reproducible research in science, as well as similar approaches for using code notebooks in digital journalism (Leon, 2021), facilitating the retracing and reconsideration of steps taken. While the generated output is a relatively “thin” description of practice, it may nevertheless stimulate documentation and encourage critical engagements with software operations. One could, for example, edit the text file and add annotations and additional documentation of analytical steps and preliminary inferences, written information and reflections to build a more comprehensive “method map” (Gerlitz et al., 2017).

In this sense, the plugin can contribute to one side of Garfinkel's account-ability: the ability to give an account of a certain epistemic situation. Yet, obtaining information on the layout algorithm used in Gephi is not sufficient to understand how the algorithm works and renders the data to which it is applied (cf. van Geenen, 2020). The plugin was thus devised to support a move from the accessibility of the research outcomes to their “assessability.” Put differently, the Fieldnotes plugin does not aim at an unattainable “accountability by design” (Wieringa et al., 2019, p. 282–292). Rather it may serve as a prompt to attend to the steps and settings of software-based network practices. That is to say, “caring for data” (Pinel et al., 2020) includes reflecting on one's technical work with and on computational research tools and the algorithmic procedures they feature.

The plugin was not primarily envisaged as a form of technical innovation. Rather, it is intended to be an invitation to reconsider and experiment with the sociotechnical arrangements involved in making and making sense with networks in different settings. These investigations could include methodological-technical work that develops and at the same time reflects on other possible settings and operations that could be accounted for, as per Agre (1997) idea of “critical technical practice.” In other words, the choice which specifications and activity might be logged should not only be based on technical feasibility, but on their role as “epistemological affordances” (van Geenen, 2020, p. 8, 17–19), or the role they play in methodological decision-making, analytical and interpretative work, and communicative efforts conducted by practitioners.

In order to generate “thicker” descriptions of network practices, some of us developed a set of experimental observation protocols which were tested in classes at King's College London (see Table 1). In a workshop on “doing things with networks” students engaged in several exercises to draw attention to processes of making and making sense of networks. The title of the workshop alludes to Austin's work on the performativity of language and associated work in STS examining how numbers, graphs, methods, models, and code are not just neutral instruments for representing collective life, but devices involved in performing, ordering and organizing things in particular ways (cf. Austin, 1975; Mackenzie, 2005; Espeland and Stevens, 2008; Ruppert et al., 2013; Verran, 2015). The workshop sought to slow down and attend to observational and interpretive work with networks through group projects related to our student's own experience and practices—such as generating and collectively interpreting networks on app usage in the class—a take on Jacob Moreno's classic study on classroom friendships through “sociograms”—to highlight affordances and limits of networks as a way of “telling about society” (Becker, 2007). Moreover, the workshop featured exercises on “noticing how we notice,” “collective descriptions,” and “making networks public” (see Table 1). These approaches were used to encourage students to explore “critical data practice” with relational data, asking them to focus not only on representational, instrumental and cognitive dimensions but also on “affective and attentive relationships with data” (Pinel et al., 2020, p. 193). The students were asked to pay attention to how networks become meaningful and are invested with particular kinds of analytical, interpretive and narrative capacities.

**Table 1:** Observation protocols from “doing things with networks” class as part of Big Data in Culture & Society MA programme at Department of Digital Humanities, King's College London.

**Theme**	**Exercise**
“Noticing how we notice”^19^	In pairs: One person describes how they observe and make sense of network (“micro-actions”). The other person takes notes and tries to come up with an observation “protocol.”
“Collective descriptions”	In your groups: One person looks at the network and makes an observation; The next person makes another observation, building on (but not repeating) what the previous person has said; Continue until there is nothing more to say.
“Making networks public”	Make a table for all of the networks, examining: “Mood” of analysis (evidence, hidden patterns); Mode of sharing (alone, together, online, offline, social networks); Materiality (single image, images series, print, leaflet, story, installation); Publics gathered and how they are invited to make sense with the network.

## 4. Conclusion: Making networks questionable

This article is a call to respond to the increasing prominence of networks in and beyond academic research, in journalism, policy, activism and other areas, with a parallel increase in “network response-ability.” Alluding to Haraway's important work in feminist STS, the notion of “network response-ability” denotes the need to nurture our capacity as network practitioners to recognize, acknowledge, and respond to differences in how network analysis and visualization is put into practice. This includes recognition that network graphs and maps are not only expert devices, but also an increasingly prominent part of contemporary digital and visual culture. In “making networks public,” paraphrasing Latour and Weibel (2005), we should also make them publicly questionable and thus relatable.

As a contribution to developing network response-ability, we inquired into diverse communities, computational actors, infrastructures and forms of mediation involved in making and making sense of and with relational data. Inspired by, alongside feminist STS, ethnomethodology, we considered the troubles of network practices not as flaws that need solving, but as invitations to explore and situate how networks and their meaning are cooperatively constructed, and work across different settings and situations. Specifically, we have presented troubles related to (2.1) roles, uses and views of networks in journalism; (2.2) the sharing, collective exploration and interpretation of networks beyond academia; and (2.3) the development of software tools for the visual analysis of networks envisioning their applicability for different user communities and scenarios of use.

Firstly, drawing on research on journalistic network practices in the making, we found that journalists use networks in different ways in their work, applying them frequently as discovery devices and—but not necessarily—as narrative devices as well. Journalists motivate this decision posing the question of network map's representativeness of the field and its actors under investigation. Secondly, we used the case of translating our own network graphs for and with broader publics to emphasize that network practices and the resulting maps need to be considered as—borrowing from Garfinkel—cooperative practical accomplishments. That is to say, the translation of network visuals across settings requires close attention to and care for the ways in which different practitioners and publics might engage with and understand network maps, as well as the epistemic principles, methodological considerations, and exploratory and interpretative work that informed their construction. Thirdly, we discussed different perspectives on and tensions regarding the usage scenarios of the MiniVAN tool, exemplified by the question whether this tool could and should just allow for an exploratory engagement, or also an explanatory engagement with network maps. These at the same time technical, epistemological, and research-ethical considerations led to an interest in situating network practices and the sociotechnical arrangements (e.g., software tools) that sustain them. Moreover, it encouraged us to explore and devise formats to surface and stay with frictions in and through the work of diverse network practitioners, especially in interdisciplinary and trans-institutional collaborations and exchanges.

In summary, we considered network troubles as prompts to explore and understand network practices, both in relation to their sociological potential (cf. Marres, 2017; Marres and Gerlitz, 2018) and in terms of their own “social lives” as research methods (cf. Ruppert et al., 2013). Moreover, we treated the troubles that the social lives of such practices bring about as occasions to make explicit the epistemic assumptions inscribed into network tools and to attend to the cultures of interpreting and making sense of networks in various societal settings. Finally, in section 3, we briefly discussed two complementary approaches to accounting for network practices: the Gephi Fieldnotes plugin and a series of protocols for observing and documenting network practices. These approaches are presented not as general “best practices.” Rather, we understand them as encouragements to consider how network practices may be cared and accounted for. Writing this article as a contribution to critical data practice, we hope to invite further consideration of how such situating approaches and accounts might modify network practices—including through cross-disciplinary collaborations between STS researchers, media researchers, data scientists, software developers, designers, journalists, artists, activists, institutions, community groups and others.

## Author contributions

DG and JG are jointly responsible for developing the overall conceptual framing of the paper drawing on a series of calls, workshops, and activities involving all authors. DG proposed a collaboration with JG and the Public Data Lab after a visit to King's College London in July 2018 to present work on the Gephi Fieldnotes Plugin, in order to further explore ethnomethodological and feminist perspectives in approaching network practices in diverse settings and situations. This collaboration was kicked-off with the MiniVAN workshop in November 2018. DG brought in the reflections on the investigative work on the Dutch Twittersphere and on the development of the Fieldnotes Plugin. The article builds on ongoing empirical work from JG and LB on network practices in digital journalism. TV led the MiniVAN proposal, reflections from which are included in the second section, in association with MJ, AM, and JG. AM introduced the situated network practices framing as a re-orientation for the MiniVAN project. AM and JG co-led the doing things with networks classes at KCL in which the various teaching protocols were tested. All authors contributed to the article and approved the submitted version.

## Footnotes

^1^According to data from Google Scholar on July 18, 2022. Notably, studies that made use of Gephi cited this paper instead of the tool itself, which also resulted in the granting of the Test of Time Award at ICWSM 2019.

^2^The diversity of practitioners was, for instance, indicated by a small-scale survey held by the Gephi development team (Bastian, 2016).

^3^The Public Data Lab explores what difference the digital makes in attending to public problems. A list of associated centers and researchers is available at: https://publicdatalab.org/.

^4^Interviews, workshops and correspondence with journalists by Gray and Bounegru, 2014-2019, including from Panama Papers, ICIJ, Reuters, Global Witness, The Guardian, The Times, the Financial Times, OCCRP, Washington Post, Influence Mappers, J++, Le Monde, Pro Publica, ICFJ, New York Times, Center for Public Integrity, Groene Amsterdammer, LA Times and Associated Press.

^5^While these issues are not specific to working with relational data, we include them in order to highlight the range of different actors, devices, and infrastructures involved in network practices.

^6^This research was conducted in collaboration with the University of Applied Sciences Utrecht and in particular the Journalism Studies research group, “Quality Journalism in Digital Transition.”

^7^For a detailed description of and reflection on the sampling methodology developed and applied see van Geenen et al. (2016).

^8^The team of researchers consisted of media scholars with backgrounds in cultural studies and social theory and expertise in digital methods, as well as a computer scientist with training in the humanities.

^9^This collection of tweets was not limited to retweets, but we broadened our sampling scope and included all kinds of tweets containing media references (hyperlinks).

^10^To establish a baseline sample, we compiled and encoded not just respectively 500 randomly chosen tweets sent by accounts in the two selected clusters, but also 500 randomly selected tweets sent by accounts in the entire retweet network.

^11^Only some of the users in the light blue and biggest community 146 could be clearly identified as politically left-wing. Moreover, this community is more scattered and heterogenous than the right-wing community 56.

^12^Departing from this assumption, Vrij Nederland presented their own calculations stating that approximately 0.01 percent of the Dutch Twitter users – the group of angry Twitter users, as they framed it – is determining and capturing the debate (Broer and Ostendorf, January 24, 2018). The paper version of the published article was even covered by a title containing this declaration.

^13^More on the “life” of one of the Dutch Twittersphere visuals can be found in van Geenen and Wieringa (2020), in which we reflect on our efforts to engage broader publics around our research using these methods, including people without hands-on experiences with network analysis or visualization. See also Grandjean and Jacomy (2019) for strategies on how to take care of such methods, when they are translated into other disciplines than the ones in which they were developed.

^14^For more information on the workshop see: https://archive.md/Ni3GF. The name of the tool MiniVAN combines the abbreviation VAN which stands for “Visual Analyzer of Networks” with the prefix “mini,” which alludes to aspirations of portability, convenience and ease of use on the web.

^15^For an elaborate list of the tools see: http://tools.medialab.sciences-po.fr/.

^16^Such as Tableau, DataWrapper, Flourish, Carto, Infogram and others.

^17^See, e.g., https://gephi.wordpress.com/2018/11/01/is-gephi-obsolete-situation-and-perspectives/ and https://gephi.wordpress.com/2019/02/02/exploring-the-dystopian-future-of-a-javascript-gephi/.

^18^See the following blogpost on the website of the SAGE Ocean Initiative for a detailed introduction of the notion of “situated network practices”: SAGE Ocean (2019).

^19^An exercise inspired by what Anna Tsing characterizes as the “arts of noticing” (Tsing, 2015).
